# Experimental manipulation of muscularity preferences through visual diet and associative learning

**DOI:** 10.1371/journal.pone.0255403

**Published:** 2021-08-11

**Authors:** Katy Jacques, Elizabeth Evans, Lynda Boothroyd

**Affiliations:** 1 Department of Psychology, Durham University, Durham, United Kingdom; 2 Department of Psychology, Newcastle University, Newcastle Upon Tyne, United Kingdom; Universite de Poitiers, FRANCE

## Abstract

Body preferences are somewhat flexible and this variability may be the result of one’s visual diet (whereby mere exposure to certain bodies shifts preferences), associative learning mechanisms (whereby cues to health and status within the population are internalised and affect body preferences), or a mixture of both visual diet and associative learning effects. We tested how these factors may drive changes in preferences for muscularity in male bodies across a male and female sample. Three studies were conducted where participants viewed manipulation images of high and/or low muscle mass males which were either aspirational (high status clothing and posture) and/or neutral (no obvious cues to status). Preferences for muscularity were recorded before and after exposure to such manipulation images to assess whether body preferences had changed following manipulation. We found evidence for both the visual diet and associative learning hypotheses. Exposure to non-muscular male bodies decreased preferences for muscular bodies irrespective of image valence. Exposure to aspirational non-muscular male bodies alongside neutral muscular male bodies also led to a decrease in muscularity preferences. Further, when manipulation conditions are less obviously skewed towards a particular body type, preferences still shifted in the direction of the most prevalent body type, suggesting that demand characteristics are unlikely to have confounded results of previous adaptation experiments with more obvious manipulations.

## Introduction

Body ideals or body preferences–that is the tendency for people to consider particular sizes and shapes of human bodies to be more attractive, appealing or desirable than others–are important in many respects. Body weight can affect an individual’s chances of social success [1], can be an important contributor to perceived attractiveness [2], and is a critical component in body image and thus a key facet of self-esteem [3]. The bulk of research on body ideals has concentrated on weight or body mass index (BMI; height in m2/weight in kg) in women, and has therefore neglected muscularity as an important body ideal in men. The current paper seeks to test mechanisms of variation in observers’ preferences for muscularity in male bodies.

### Drivers of variation in preferences

The extensive variability in body size and shape preferences is particularly evident from cross cultural work, although it largely concentrates on female BMI as noted Research participants in Western, industrialised cultures, with a reliable food supply, for example, prefer thinner female figures, while some non-Western populations, with unreliable food supplies, prefer larger female bodies e.g. [4, 5]. Variability also exists within ethnic groups; for instance urban Thai participants associate high BMI with low health and fertility, while the converse is true in rural Thailand, resulting in different body size preferences [6]. A similar association between body preferences and socio-economic development was documented in Malaysia [7].

In the Western world (meaning predominantly Europe, and other White-majority countries), thinner women are mostly viewed in a positive light, with people more willing to engage in social, academic and recreational activities with these individuals [8], whilst overweight female figures are often stigmatised [9]. Higher body mass (indexed by obesity) is increasingly associated with lower SES within countries (e.g. Hispanic and white youth US samples [10]). This, together with the fact that malnourishment is extremely rare in the West, suggests that amongst Westernized samples low BMI female bodies are associated with perceptions of better health, higher prestige and higher SES, whilst high BMI female bodies often have negative associations. Researchers propose that the thin ideal is starting to become widely international in nature and that this, at least in part, can be explained as a function of globalisation of Western media [11]. This may also explain why body preferences appear to move towards Western body ideals when, for example, South Africans migrate to Britain [5]; they begin to adopt the thin ideal that is so prevalent in the UK. Similarly, evidence suggests non-Western individuals who consume Western media show changes in body size preferences towards lower BMI for females [11–13] and experimentally viewing idealised bodies increases preferences for low BMI female bodies in laboratory studies [14–17].

Boothroyd and colleagues [17] argued that there are two potential routes through which globalisation of media may influence preferences. Firstly, individuals and larger groups may vary in ‘visual diet’, with high (or low) levels of exposure to a particular category of stimulus (in this instance very slim women in media) inducing changes in preferences through visual adaptation effects altering the individuals’ perceptions of a ‘normal’ example of that stimulus. Secondly, given the associations with body weight discussed above, ‘simple’ associative learning mechanisms may also play a role [17]. They therefore sought to explore the underlying internal mechanisms underpinning changes in preferences for BMI in female bodies using visual adaptation procedures and manipulation images that varied in BMI and valence. They found evidence for the visual diet hypothesis, with exposure to thin or large bodies shifting participants’ body weight preferences in the predicted direction, regardless of whether or not bodies were aspirational or non-aspirational. The authors also carried out a further study as part of the same paper which induced associative learning whilst making a visual diet effect impossible (equal numbers of large and thin images of varying valence). Exposure to overweight, aspirational women, together with equal exposure to low BMI, non-aspirational women, resulted in a shift in body weight preferences towards larger bodies. Overall findings thus suggested both visual diet and associative learning influences may act, to an extent, in parallel. However, consistent with the previous body preference literature, this study did not examine changes in preferences for muscularity in male bodies [17].

### Preferences for male muscularity

As already noted, female bodies have been used as stimuli in the vast majority of research on variation in body size ideals. Preferences for male muscularity and variability in such preferences has been an understudied area but is worthy of consideration. The limited existing male body literature suggests that preferences for muscularity in males may, like preferences for BMI in females, be variable across cultures. For example, when asked to rate individual photographs of men whose bodies varied in waist-to-chest-ratio (WCR), body-mass-index (BMI) and waist-to-hip-ratio (WHR), men in urban settings in both Britain and Kuala Lumpur preferred slim male bodies with the muscular ‘inverted triangle’, over those bodies with higher body mass and thus a less pronounced upper body shape. This suggests WCR as the primary component of attractiveness for these individuals. Conversely, in the rural region of Kota Belud, in Sabah, one of East Malaysia’s least economically developed states, men preferred heavier bodies with a less triangular shape and BMI was statistically the primary predictor of male body attractiveness [18].

Media also often over-represent idealised, muscular male bodies [19, 20] just as they do slim female bodies. It is therefore likely that frequent exposure could shift perceptions of normality and preferences towards male muscularity. To date, one study has observed experimental visual adaptation to muscularity in the laboratory [21].

Furthermore, muscularity may also, like slimness, be associated with health and high status in the Western culture. For instance, favourable stereotypes of muscular male bodies have been observed, with participants describing them as physically healthy, clean and attractive. In contrast, they possess negative stereotypes of non-muscular endomorphs, describing them as physically unhealthy, dirty and unattractive [22]. Western figures in media are often of high SES and are associated with positive attributes, for example, muscular figures in top-grossing films between the years of 1980–2006 are more likely to be central characters who are romantically involved with others, experience more sexual activity and who experience more positive outcomes in such films [23].

Because muscular male figures now dominate much of Western media [19, 20], are frequently positively valenced (19), and are frequently digitally manipulated to further enhance muscular bodily features [24], it is likely such media exposure is affecting our body preferences in the real world, just as such media exposure increases preferences for idealised body types [17] and affects ‘perception of normality’ [15, 25] in female bodies. Indeed, it is critical to focus more research on this understudied area of male muscularity preferences, investigating whether viewing idealised male media imagery can affect conceptions of what such males subsequently view as ‘ideal’ or ‘normal’ standards for their own bodies. Further, exploring potential mechanisms underpinning changes in these preferences is crucial if we are to work towards developing successful strategies aimed at improving male body image in the West.

### Current study

The current research aimed to build upon the methods of Boothroyd et al [17] to investigate whether shifts in preferences for muscularity are the result of our ‘visual diet’ (the idea mere exposure to certain body shapes can shift our preferences); due to ‘associative learning’ mechanisms (the idea that muscularity is associated with positive attributes of health and status in the west), or a mixture of both. This paper explores changes in preferences for muscularity in male bodies across both male and female observers. We include female participants in our sample as both men and women are exposed to media messaging related to what is attractive/normative/ high status in a male body, so we would argue that it is reasonable to expect that both men and women’s preferences should be affected by increased exposure to males of a particular body type and valence. Indeed previous work on adaptation to muscularity e.g. [21], found effects for both men and women viewing both male and female stimuli. That study, however, used neutral stimuli (e.g. images of males in standardised tight fitting grey singlets and shorts, posed in a standardised anatomical position) and thus failed to explore whether susceptibility to visual adaptation is more likely when stimuli used are of a positive valence (e.g. males in high status clothing, with high status posture of differing muscle mass), something that the current study seeks to address. In Study 1, manipulation conditions involved participants viewing either aspirational, high muscle mass male bodies (Condition 1), aspirational, low muscle mass bodies (Condition 2), neutral, high muscle mass bodies (Condition 3) or neutral, low muscle mass bodies (Condition 4). We predicted that under the visual diet hypothesis, exposure to muscular (non-muscular) male bodies would cause a shift in preference towards muscular (non-muscular) male bodies irrespective of whether or not that image was of a positive or neutral valence. However, under the associative learning hypothesis, we predicted that only those muscular (non-muscular) male bodies of a positive valence would shift preferences towards a more muscular (non-muscular) male body type. If the evidence pointed towards a visual diet mechanism, this would imply that media’s over-emphasis of muscular male bodies has potentially shifted body preferences and perhaps our perceptions of a normal male body. However, if the evidence pointed towards associative learning mechanisms, this would imply that changes in the preferences for muscularity amongst males and females may be explained by the internalisation of positive associations attributed to muscularity in the West (e.g. health and high-status) and this could be due to the way in which media portrays high muscle mass males. Based on previously cited similar work [17], we hypothesise that the viewing of high (low) muscle mass images may too increase (decrease) preferences for muscularity and this change will potentially be stronger when images are of a positive valence.

We ran two further manipulation conditions (Study 2), to explore whether associative learning effects could be observed in a situation in which visual diet effects would be impossible to observe. Study 2 involved participants viewing either a combination of aspirational high muscle mass male bodies together with neutral low muscle mass male bodies (Condition 5), or, viewing a combination of aspirational low muscle mass male bodies together with neutral high muscle mass bodies (Condition 6). Specifically, we are testing the hypothesis that preferences for muscularity are likely to shift in the direction of the high valence image type, whether it be high or low muscle mass.

A key consideration in studies using repeated exposure to similar stimuli is that of demand characteristics i.e. participants discerning the intention of the study and consciously shaping their responses in line with this. In line with the recommendations of others e.g. [26], we include a study that made use of ‘distractor images’ presented alongside the idealised manipulation images of bodies to lessen the likelihood of demand characteristics acting as a confound and test whether adaptation still occurs when manipulation image bias towards a particular body type is more subtle. In Study 3, therefore, participants viewed manipulation images that consisted of 69% high muscle mass images versus 31% low muscle mass images or vice versa. We then measured whether there was a change in preference for muscularity under each of these manipulation conditions. If the adaptation effect holds, we predict that preferences for muscularity will still change in the direction of the most prevalent image type viewed (high or low muscle mass). Pre-existing body concerns, in both Studies 1 & 2, was measured using the Drive for Muscularity Scale (DMS) [27]. Evidence from research with women suggests pre-existing concerns such as body dissatisfaction may moderate how susceptible one is to visual adaptation effects, with most research (with the exception of [17]) finding that individuals who are dissatisfied with their bodies are more susceptible to visual adaptation effects in the body size dimension [15, 25]. As an exploratory part of our research, we sought to explore whether the same may be true of males in our sample when assessing susceptibility to the visual adaptation effect in the muscularity dimension. Specifically, we hypothesise that those who are most concerned about the muscularity of their bodies (as measured by DMS) are those who will show stronger shifts in their preferences for muscularity following manipulation.

## Study 1

### Method

#### Ethics

Ethical approval for all three studies was gained from Durham University’s Psychology Department Ethics Committee. Participants provided online consent before the trials began by clicking a box to confirm they had read and understood the participant information sheet and privacy notice. Participants were shown the debrief statement on screen once they had completed all trials and were provided with a web link to a popular body image support website.

#### Participants

An *a priori* power analysis was conducted to determine the minimum number of participants required. We based this analysis on the interaction effect (time by condition) in a study of a very similar design [17] (where they found a significant interaction between test phase and model size (*F*_1,52_ = 23.397, *p*<0.001, partial eta^2^ = 0.310)), with alpha set to 0.05 and power set to 0.8. This power analysis revealed a sample size of at least 92 participants was required to test for a 3-way interaction in Study 1.

The study was conducted remotely online via Qualtrics and participants were recruited from the university’s departmental participant pool, word of mouth and snowball sampling. Participants were entered into a £50 prize draw as thanks for their time and received course credits for their participation where appropriate. 190 (74 male and 116 female) participants were recruited, with most participants (63 men and 86 women) selecting the 18–24 and 25–30 age categories. Most participants (66 men and 102women) reported that their sexual orientation was ‘heterosexual’. The study was listed on the University’s participant pool page until recruitment naturally came to a standstill. Participants were randomly allocated to one of four manipulation conditions, counterbalanced on the basis of their birth month (e.g. January, May, September = Condition 1; February, June, October = Condition 2; March, July, November = Condition 3; April, August, December = Condition 4). Participants were told that the aim of the experiment was to explore ‘body preferences’. On average the study took 12.7 minutes for participants to complete.

#### Preference for muscularity task

The preference stimuli (12 CGI images of male bodies varying in muscle mass) were created using DAZ Studio 4.10, using the ‘Genesis 2 Base Male’ in basic white briefs. 6 high muscle mass and 6 low muscle mass versions of this body with identical faces were created in total. The 6 muscular male images had the built in ’Bodybuilder’, ’Bodybuilder Details’ and ’Bodybuilder size’ slider settings set to either medium or high and the 6 non-muscular male images had these set to low as well as the ’Emaciated’ slider setting set to either medium or high. Each high muscle mass CGI image was randomly paired with a low muscle mass CGI image, creating 6 trials in total.

**Fig 1:**
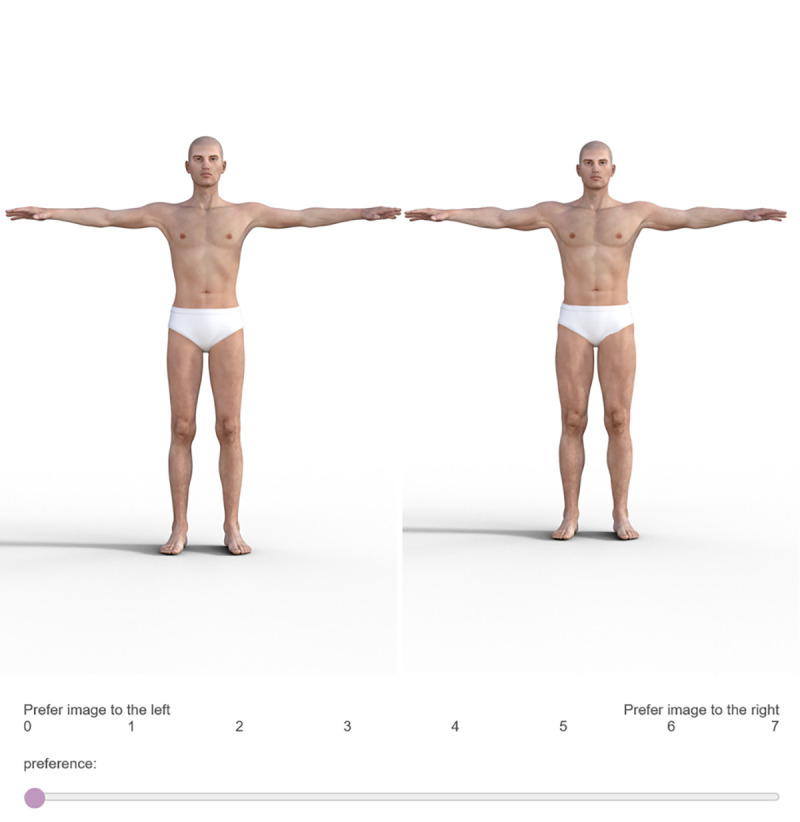
Example trial from the pre- and post- manipulation preference task.

After reporting their age, gender and sexual orientation, participants were presented with 6 pairs of CGI images (presented one pair at a time) and were asked to indicate which image from each pair they preferred and the extent to which they preferred it using an 8 point slider scale from ‘0 strongly prefer left body ‘ (low muscle mass body) to ‘7 strongly prefer right body’ (high muscle mass body), with the muscular body presented to the right hand side for half of all trials and the left hand side for the remaining trials in a randomised order. An example trial from the preference task is presented in Fig 1. Overall muscularity preference scores for the pre-manipulation preference task were generated by averaging the preference scores for each of the 6 trials. A high average score indicated a preference for high muscle mass male bodies and a low score indicated a preference for low muscle mass male bodies. Participants were asked to complete this preference task again following the manipulation phase to assess whether their preference for muscularity had changed following manipulation.

#### Manipulation phase

Participants were told the manipulation phase of the study was designed to further explore body preferences. They were shown a series of images (presented individually) and were asked to compare each new image presented to the image seen in the preceding trial whilst indicating which one they found the most attractive (for the first image presented, participants were asked to compare it to the last viewed pre-manipulation preference phase image). The order of presentation was randomised and the purpose of asking participants to indicate preferences during this phase was simply to keep participants focused on the stimuli.

47 (19 male and 28 female) participants were allocated to Condition 1 and viewed 50 aspirational high muscle mass male bodies. 44 (16 male and 28 female) participants were allocated to Condition 2 and viewed 50 aspirational low muscle mass male bodies. 45 (18 male and 27 female) participants were allocated to Condition 3 and viewed 48 neutral high muscle mass male bodies. 54 (21 male and 33 female) participants were allocated to Condition 4 and viewed 48 neutral low muscle mass male bodies.

The aspirational manipulation images (Conditions 1 and 2) were photographs of attractive, high muscle mass (Condition 1) and low muscle mass (Condition 2) males in high status clothing and in high status postures from various male clothing websites (e.g. Father Sons Clothing and Fred Perry). Neutral manipulation stimuli were open-access images retrieved from [28]. These neutral images consisted of 24 high muscle mass (Condition 3) and 24 low muscle mass (Condition 4) photographs of nude males, with bodies in a standard anatomical position (standing with arms out to the side, legs apart and facing the camera straight on) with faces and genitals obscured. Each of the neutral images were presented twice (once in normal alignment and once in mirror image version) to create a total of 48 images each for both Condition 3 and 4. All manipulation images fell under the fair use consideration of copyright legislation at the time of study. All manipulation images were pre-rated for muscularity (on a scale of 0–10) using a sample of 15 18 year old students (6 males and 9 females) and were then grouped accordingly (≥6/10 = high muscle mass image and ≤4/10 = low muscle mass image).

Following the manipulation phase, participants were told that they needed to complete the second half of the preference task. This involved completing the same preference task as was required during the pre-manipulation preference for muscularity task.

#### Muscle concerns

Following the post-manipulation preference for muscularity task, participants completed the Drive for Muscularity Scale (DMS) [27], a 15 item, self-report measurement in which participants indicate the extent to which a series of attitudes and behaviours are descriptive of themselves. Every item is scored on a Likert-type scale from 1 (Always) to 6 (Never) with scores reverse coded before summing responses. The 15 items are made up of 7 attitudinal items, for example, ‘I wish that I were more muscular’, 1 behavioural item, ‘I think about taking anabolic steroids’ and 7 combined attitudinal and behavioural items, for example ‘Other people think that I work out with weights too often’. Following this final phase of the study, participants were thanked for their participation and shown the debrief statement.

### Results

Descriptive statistics for all variables in each gender and condition are presented in Table 1 below.

**Table 1:** Tabulated mean (standard deviation) pre- and post-manipulation preference for muscularity scores, and total drive for muscularity scale scores (DMS) for each gender across the four manipulation conditions.

Condition	Gender	Mean pre-manipulation preference	Mean post-manipulation preference	Mean total DMS score
1	Male (N = 19)	5.386 (1.087)	5.483 (1.211)	49.421 (14.037)
	Female (N = 28)	4.411 (1.479)	4.756 (1.241)	31.821 (10.555)
2	Male (N = 18)	5.188(.913)	4.781 (1.018)	47.625 (13.038)
	Female (N- = 28)	4.774 (1.332)	4.601 (1.410)	26.000 (8.890)
3	Male (N = 18)	4.824 (1.235)	4.787 (1.196)	43.611 (18.363)
	Female (N = 27)	4.792 (1.326)	4.679 (1.555)	30.250(9.610)
4	Male (N = 21)	5.175 (1.031)	4.691 (1.407)	44.095 (13.490)
	Female (N = 33)	4.417 (1.487)	3.912 (1.590)	28.250 (9.873)

**Fig 2:**
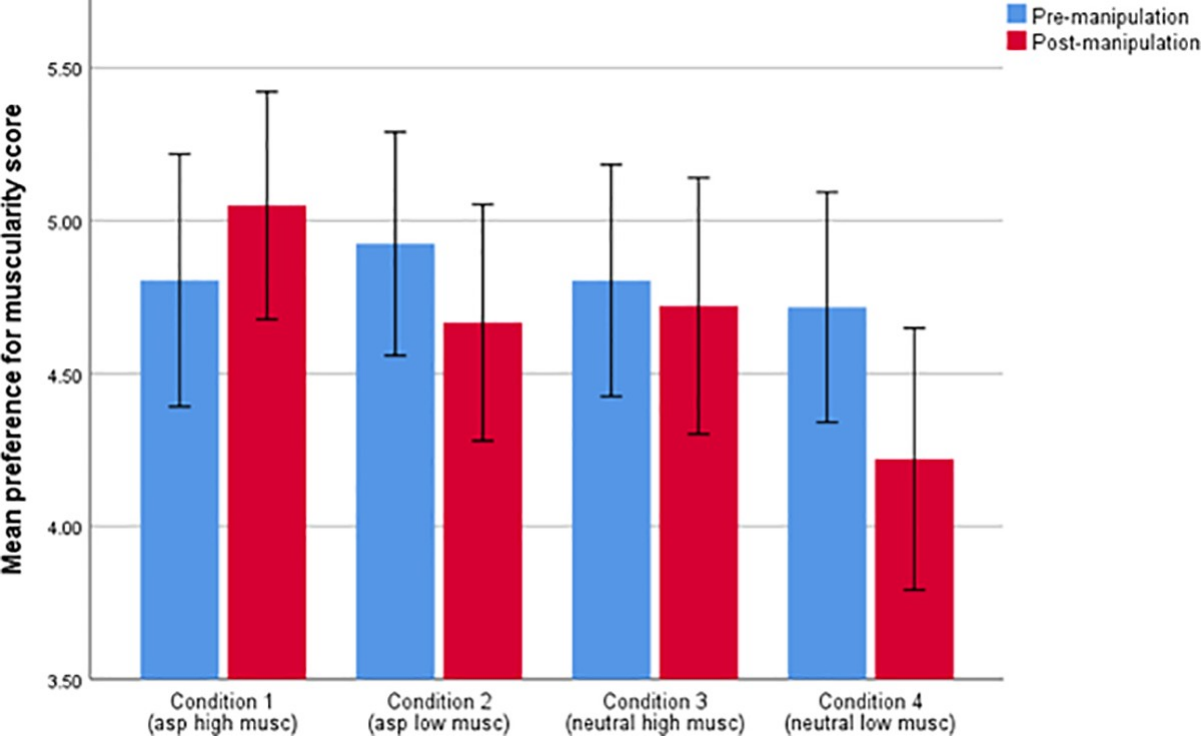
Mean preference for muscularity score for the pre- and post-manipulation preference phases for each of the 4 experimental conditions where 3.50 represents no preference to either image presented.

In order to test our main hypotheses, a mixed ANOVA was run where test phase (pre- versus post-manipulation) was a repeated measures variable, and model muscularity (high muscle mass or low muscle mass) and model valence (aspirational or neutral) were between-participant factors. Full model results are given in Table 1. As predicted by the visual diet hypothesis, there was a significant interaction between test phase and model muscularity (*F*_1,186_  =  16.646, *p*<0.001, partial eta^2^  =  .082) such that preference for high muscle mass male bodies, on average, decreased following exposure to low muscle mass manipulation images. Whilst mean preference scores increased significantly following exposure to high muscle mass aspirational manipulation images, they did not, on average, increase following exposure to the neutral high muscle mass images as shown in Table 1 and Fig 2. A post-hoc paired-samples t-test revealed a significant difference between mean pre- and post-manipulation muscularity preference scores for those viewing low muscle mass males under conditions 2 and 4 (*t*(96) = 4.658, *p* = < .000) but no such significant difference for those viewing high muscle mass images under conditions 1 and 3 (*t*(92) = -1.079, *p* = .283). The significant result for condition 2 survived when p values were corrected for multiple comparisons (using adjusted p = 0.025 for 2 tests).

In contrast to the associative learning hypothesis, there was no significant three way interaction between phase, model muscularity and model valence (*F*_1,186_  =  0.156, *p*  =  0.693, partial eta^2^  =  .001) as shown in Table 2, such that the phase and model muscularity interaction held for both aspirational and neutral manipulation conditions. As shown in Fig 2 however, although the effect of phase was more negative for participants in the low muscle mass conditions than in the high muscle mass conditions, this did not translate into participants in both high muscle mass conditions showing an increase in muscularity preferences. In fact, participants in the neutral high muscle mass condition showed no change over time.

**Table 2:** Tabulated values for mixed ANOVA with phase (pre- versus post-manipulation preference for muscularity scores) as the repeated measures variable, and model muscularity (high muscle mass or low muscle mass) and model valence (aspirational or neutral) as between-participant factors. Critical tests of our hypotheses are shown in bold.

Source	*Df*	*F*	*p*.	*ηp* ^*2*^
Phase	1, 186	6.982	0.009	0.036
Valence	1, 186	1.694	0.195	0.009
Muscularity	1, 186	1.272	0.261	0.007
Valence*Muscularity	1, 186	0.185	0.668	0.001
Phase * Valence	1, 186	6.388	0.012*	0.033
Phase * Muscularity	**1, 186**	**16.646**	<0.000**	**0.082**
Phase * Valence * Muscularity	**1, 186**	**0.156**	**0.693**	**0.001**

When gender or DMS score was added to the model, results did not change; there was still a significant interaction between phase and muscularity and there was no higher order interaction with either gender nor DMS (see Table 3 below).

**Table 3:** Tabulated values for mixed ANOVA with phase (pre- versus post-manipulation preference for muscularity scores) as the repeated measures variable, and model muscularity (high muscle mass or low muscle mass), model valence (aspirational or neutral) and participant gender (Model 1) as between-participant factors, Drive for Muscularity scale scores (DMS) added as a covariate (Model 2) or both gender and DMS added to the model (Model 3). Critical tests of our hypotheses are shown in bold.

Source	*Df*	*F*	*p*.	*ηp* ^*2*^
**Model 1**				
Phase	1, 182	7.599	0.006	0.04
Valence	1, 182	1.902	0.17	0.01
Muscularity	1, 182	1.072	0.302	0.006
Gender	1, 182	6.782	0.01	0.036
Valence*Muscularity	1, 182	0.017	0.898	0.000
Valence*Gender	1, 182	0.164	0.686	0.001
Muscularity*Gender	1, 182	0.036	0.85	0.000
Muscularity* Valence*Gender	1, 182	2.694	0.102	0.015
Phase * Valence	1, 182	4.684	0.032*	0.025
Phase * Muscularity	**1, 182**	**16.128**	<0.000**	**0.081**
Phase*Gender	1, 182	0.692	0.407	0.004
Phase * Valence * Muscularity	**1, 182**	**0.153**	**0.696**	**0.001**
Phase*Valence*Gender	1, 182	1.566	0.212	0.009
Phase*Muscularity*Gender	1, 182	0.007	0.932	0.000
Phase*Valence*Muscularity*Gender	1, 182	0.023	0.88	0.000
**Model 2**				
Phase	1, 182	1.783	.183	0.010
DMS	1, 182	33.102	<0.000	0.154
Muscularity	1, 182	0.655	0.420	0.004
Valence	1, 182	0.426	0.515	0.002
Valence* Muscularity	1, 182	2.020	0.157	0.011
Muscularity *DMS	1, 182	1.236	0.268	0.007
Valence * DMS	1, 182	0.107	0.744	0.001
Valence * Muscularity * DMS	1, 182	1.373	0.243	0.007
Phase*DMS	1, 182	0.218	0.641	0.001
Phase * Muscularity	**1, 182**	**8.218**	0.005**	**0.043**
Phase * Valence	1, 182	7.829	0.006**	0.041
Phase * Muscularity * Valence	**1, 182**	**0.146**	**0.703**	**0.001**
Phase* Muscularity * DMS	1, 182	1.942	0.165	0.011
Phase* Valence *DMS	1, 182	3.816	0.052	0.021
Phase*Valence*Muscularity* DMS	1, 182	0.024	0.876	0.000
**Model 3**				
Phase	1, 178	3.938	0.049	0.022
DMS	1, 178	20.838	<0.000	0.105
Muscularity	1, 178	0.679	0.411	0.004
Valence	1, 178	0.338	0.562	0.002
Gender	1, 178	0.984	0.322	0.006
Muscularity*Gender	1, 178	0.772	0.381	0.004
Valence*Gender	1, 178	0.200	0.656	0.001
Muscularity*DMS	1, 178	1.861	0.174	0.010
Valence * DMS	1, 178	0.214	0.644	0.001
Muscularity * Gender *DMS	1, 178	1.540	0.216	0.009
Valence * Gender * DMS	1, 178	0.302	0.583	0.002
Phase*Muscularity	**1, 178**	**7.919**	0.005*	**0.043**
Phase * Valence	1, 178	3.836	0.052	0.021
Phase * Gender	1, 178	0.956	0.330	0.005
Phase * DMS	1, 178	2.777	0.097	0.015
Phase*Muscularity*Gender	1, 178	0.319	0.573	0.002
Phase*Valence*Gender	1, 178	0.464	0.497	0.003
Phase * Muscularity * DMS	1, 178	2.744	0.099	0.015
Phase * Valence *DMS	1, 178	2.137	0.146	0.012
Phase* Muscularity * DMS * Gender	1, 178	0.013	0.911	0.000

### Study 1 interim discussion

Study 1 aimed to explore the mechanisms underpinning changes in preferences for muscularity across a Western sample. Four manipulation conditions were created to assess the extent to which visual diet or associative learning mechanisms best explained such changes in body preferences. Overall, the findings provide evidence for the visual diet hypothesis for low muscle mass images in particular.

Under the visual diet hypothesis, viewing high (versus low) muscle mass male bodies should cause a shift in preference towards higher muscle mass male bodies irrespective of whether or not that image is of a positive or neutral valence. The current findings somewhat support this prediction as exposure to low muscle mass males decreased later preferences for muscularity irrespective of whether male bodies were of a positive or neutral valence. Exposure to high muscle mass males increased preferences for muscularity under some circumstances (i.e. when these images were of a positive valence as in Condition 1) but not others (i.e. when these high muscle mass males were of a neutral valence, as in Condition 3, exposure to such males did not increase preferences for muscularity). One may explain such findings in the context of associative learning mechanisms; the stimuli used in Condition 3 were of a neutral rather than positive valence and, according to the associative learning hypothesis, neutral males should have little to no effect on one’s later preference for muscularity. However, this fails to explain why the neutral images, used in Condition 4, changed preference for muscularity so drastically (a bigger change in preference than any of the changes observed under the aspirational conditions). Indeed, we found no significant three-way interaction between phase, muscularity and valence, such that the interaction between phase and muscularity held for both aspirational and neutral images.

When gender was added as an additional predictor, there was still a significant interaction between phase and muscularity but there were no higher order interactions (see Table 3), suggesting that males and females are equally prone to visual diet effects. Further, consistent with other research where body dissatisfaction had no effect on weight adaptation effects in women [17], the exploratory analyses of Study 1 suggest a participant’s Drive for Muscularity score does not affect how likely they are to change their muscularity preferences following exposure to males of either high or low muscle mass. This goes against findings of some of the previously cited work by [15, 25], who noted that participants with pre-existing concerns were more susceptible to visual adaptation effects in the body size dimension. We will consider the possible explanations of this null effect as part of our later discussion.

Study 1 shows good support for the effects of visual diet for low muscle mass images, but associative learning may still take place in circumstances where visual diet is not in effect [17]. With this in mind, we conducted a second study in which visual diet effects were impossible, yet associative learning effects could still arise. Specifically, Study 2 explored whether exposure to an equal number of aspirational high muscle mass and neutral low muscle mass images (Condition 5) decreased or increased preferences for muscularity, and, whether exposure to an equal number of aspirational low muscle mass and neutral high muscle mass images (Condition 6) decreased or increased preferences for muscularity.

## Study 2

### Method

#### Participants

For Study 2, we aimed to exceed the number of participants recruited in a previous study of very similar design (e.g. [17]) and that of previous muscularity adaptation work (e.g. [21]). We therefore recruited 84 (31 men and 53 women) participants with a mean age of 31 (SD = 12.06) and this exceeds the number of participants required for a two-way interaction using the power analysis that we ran for Study 1. Participants were recruited through the university’s departmental participant pool, word of mouth and snowball sampling. 89% of the sample reported that they were exclusively heterosexual. Prior to the pre-manipulation preference for muscularity task, participants were randomly allocated to one of two manipulation conditions. Participants were told that the aim of the experiment was to explore ‘body preferences’. On average the study took 9 minutes and 30 seconds for participants to complete. Participants were entered into a £50 prize draw as a thank you for their time.

#### Stimuli

Stimuli from Study 1 were pre-rated for muscularity and valence in order to select the most appropriate stimuli for Study 2. Thirty-two (6 male and 26 female) 18-year-old participants (all but one exclusively heterosexual) responded to the pre rating survey. Participants were asked to ‘Rate each image in terms of whether you think men would aspire to be like this person (e.g. in style and status) from 0 (I don’t think men would at all aspire to be this person) to 10 (men would definitely aspire to be like this person)’ and to ‘Rate each image in terms of how muscular you find the body from 0 (not at all muscular) to 10 (extremely muscular)’. Using these data we selected the most appropriate images to use in Study 2 (24 aspirational high muscle mass images, 24 neutral low muscle mass images, 24 aspirational low muscle mass images and 24 neutral high muscle mass images). The mean ratings for valence and muscularity across each of these stimuli categories are presented in Table 4.

**Table 4:** Results from the pre-rating task: Mean ratings for valence and muscularity for each of the 24 selected images across each of the 4 stimuli categories.

Stimuli for Study 2	Aspirational high muscle (Condition 5)	Neutral low muscle (Condition 5)	Aspirational low muscle (Condition 6)	Neutral high muscle (Condition 6)
Mean valence rating (max = 10)	7.590	2.648	5.268	4.708
Mean muscularity rating (max = 10)	7.337	2.272	2.906	4.840

#### Procedures

Procedures matched those described for Study 1, with the only change being to the manipulation conditions. In Study 2 participants were randomly assigned to one of two manipulation conditions. Condition 5 involved 44 (19 male and 25 female) participants viewing 24 aspirational high muscle mass male bodies and 24 neutral low muscle mass male bodies. Condition 6 involved 40 (12 male and 28 female) participants viewing 24 aspirational low muscle mass male bodies and 24 neutral muscular males. All manipulation images were presented in a randomised order.

### Results

Descriptive statistics outlining the average pre- and post- manipulation preferences for muscularity scores, and average total DMS scores, by gender and condition are presented in Table 5.

**Table 5:** Tabulated mean (standard deviation) pre- and post-manipulation preference for muscularity scores, and total DMS scores for each gender across the two manipulation conditions.

Condition	Gender	Mean pre-manipulation preference	Mean post-manipulation preference	Mean total DMS score
5	Male (N = 19)	4.833 (1.442)	5.053 (1.115)	44.211 (15.747)
	Female (N = 25)	4.760 (1.276)	4.480 (1.342)	30.160 (8.275)
6	Male (N = 12)	5.139 (.819)	4.528 (1.216)	41.917 (10.396)
	Female (N = 28)	4.738 (1.393)	4.268 (1.739)	31.357 (12.284)

**Fig 3:**
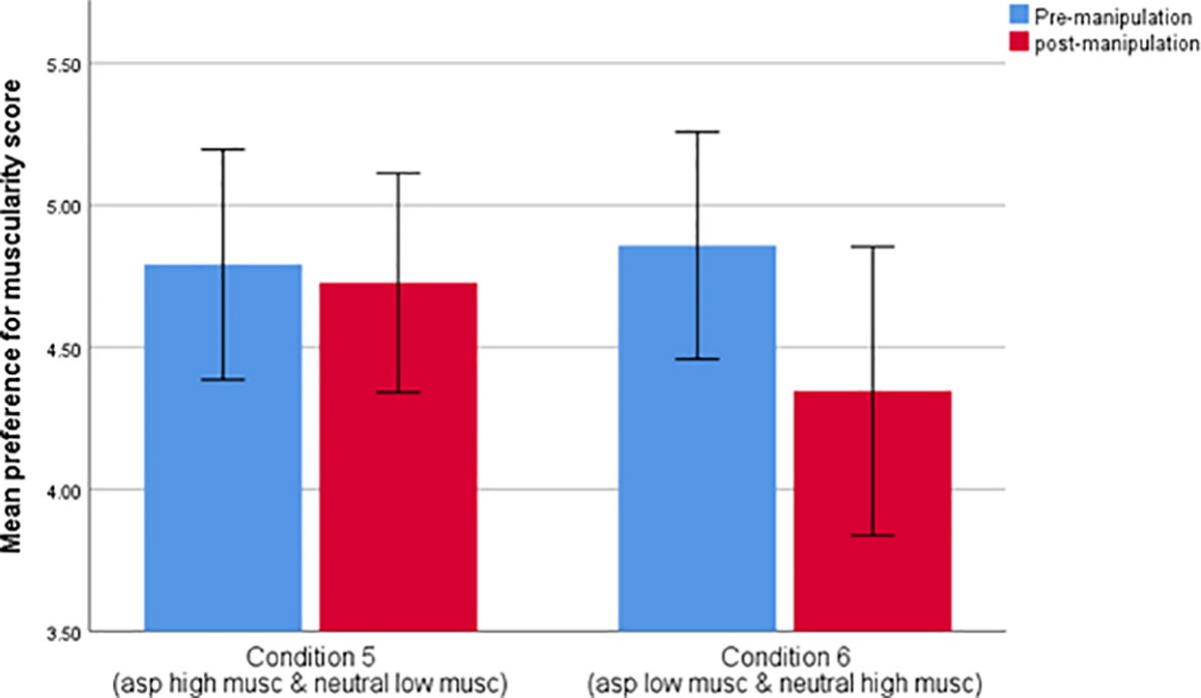
Mean changes in preference for muscularity following manipulation across each of the two conditions where 3.50 represents no preference to either image presented.

A mixed ANOVA with test phase (pre- versus post-manipulation) as a repeated measures variable and condition (Condition 5 versus Condition 6) as the between-participant factor showed a significant interaction between test phase and condition (F_1,82_ = 8.690, *p* < .005, partial eta^2^ = .096) such that Condition 6 manipulation stimuli (aspirational non muscular and neutral muscular images) decreased preferences for muscularity to a greater extent than the Condition 5 manipulation stimuli (made up of aspirational muscular and neutral non-muscular images). Mean post-manipulation changes in muscularity preference for each of the two conditions are presented in Fig 3 and the tabulated values for the mixed ANOVA are shown in Table 6. A post-hoc paired-samples t-test revealed a significant difference between mean pre- and post-manipulation muscularity preference scores for condition 6 (*t*(39) = 4.621, *p* = < .000), but not for condition 5 (*t*(43) = .618, *p* = .540).

**Table 6:** Tabulated values for mixed ANOVA with phase (pre- versus post- manipulation preference for muscularity scores) as the repeated measures variable and condition (condition 5 versus condition 6) as the between-participants factor. Critical tests of our hypotheses are shown in bold.

Source	*Df*	*F*	*p*.	*ηp* ^*2*^
Phase	1, 82	14.403	<0.000	0.149
Condition	1, 82	0.298	0.587	0.004
Phase * Condition	**1, 82**	**8.69**	0.004**	**0.096**

**Fig 4:**
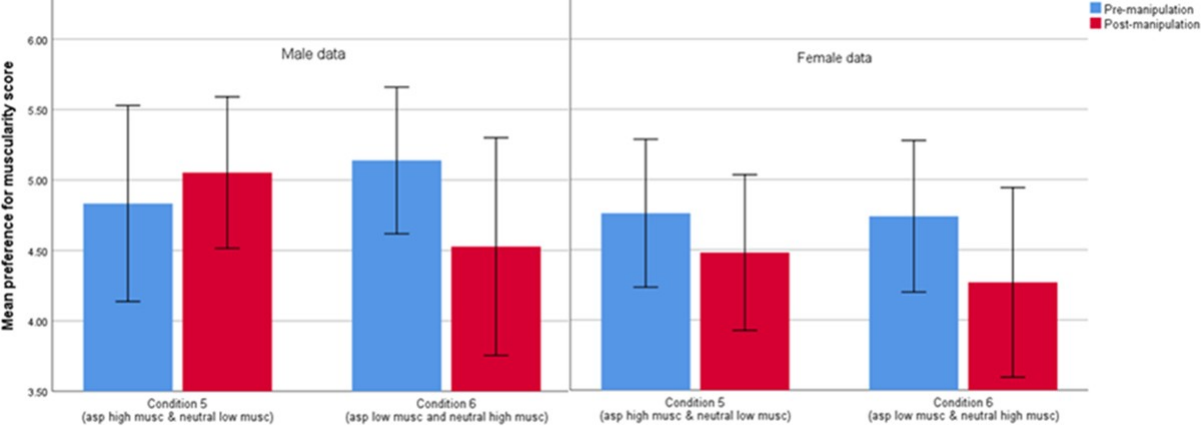
Mean preference for muscularity score for the pre- and post-manipulation preference phases for each of the 2 experimental conditions split by gender where 3.50 represents no preference to either image presented.

Further analyses also revealed a three-way interaction between phase (pre-versus post-manipulation preference score), condition and gender (F_1,80_ = 4.204, *p* < .045, partial eta^2^ = .050) as shown in Table 7. Gender differences in muscularity preference score, pre- and post-manipulation for both conditions are shown Fig 4.

**Table 7:** Tabulated values for mixed ANOVA with phase (pre- versus post- manipulation preference for muscularity scores) as the repeated measures variable, and condition (condition 5 versus condition 6) and gender (male versus female) as between-participant factors. Critical tests of our hypotheses are shown in bold.

Source	*df*	*F*	*p*.	*ηp* ^*2*^
Phase	1, 80	13.379	0	0.143
Condition	1, 80	0.138	0.711	0.002
Gender	1, 80	1.147	0.287	0.014
Condition*Gender	1, 80	0.000	0.99	0.000
Phase*Condition	**1, 80**	**10.686**	0.002**	**0.118**
Phase*Gender	1, 80	1.318	0.254	0.016
Phase*Condition*Gender	1, 80	4.204	0.044*	0.050

When male and female data were split and analysed separately the mixed ANOVA analysis for females showed no significant interaction between test phase and condition (F_1,51_ = 1.000, *p* = .322, partial eta^2^ = .019). However, the analysis for male data did show a significant interaction between phase and condition (F_1,29_ = 11.799, *p* < .003, partial eta^2^ = .289). Paired-sample t-tests revealed a significant difference between mean pre- and post-manipulation muscularity preference scores for condition 6 (*t*(11) = 2.916, *p* = .014) but not condition 5 (*t*(18) = 1.570, *p* = .134).

A further mixed ANOVA analysis on combined male and female data, with DMS score added to the model, revealed no significant interactions (and indeed the main interaction of interest become marginal). Tabulated values for the male data mixed ANOVA are presented below in Table 8.

**Table 8:** Tabulated values for mixed ANOVA with phase (pre- versus post-manipulation preference for muscularity scores) as the repeated measures variable and condition as the between-participants factor and DMS as a covariate for male data. Critical tests of our hypotheses are shown in bold.

Source	*df*	*F*	*p*.	*ηp* ^*2*^
Phase	1, 27	0.272	0.606	0.010
DMS	1, 27	6.179	0.019	0.186
Condition	1, 27	0.129	0.722	0.005
Condition*DMS	1, 27	0.142	0.709	0.005
Phase*DMS	1, 27	0.897	0.352	0.032
Phase*Condition	**1, 27**	**3.211**	**0.084**	**0.106**
Phase*Condition *DMS	1, 27	0.758	0.392	0.027

A mixed ANOVA analysis with test phase (pre- versus post-manipulation) as a repeated measures variable, DMS score, condition (Condition 5 versus Condition 6) and gender as the between-participant factors revealed a significant interaction between phase and condition (F_1,76_ = 4.483, *p* = < .039, partial eta^2^ = .056) but no other significant interactions. Findings are presented in Table 9. Further, no significant interactions were found when male and female data were isolated and analysed separately.

**Table 9:** Tabulated values for mixed ANOVA with phase (pre- versus post-manipulation preference for muscularity scores) as the repeated measures variable, and condition and participant gender as between-participant factors with DMS as covariate. Critical tests of our hypotheses are shown in bold.

Source	*Df*	*F*	*p*.	*ηp* ^*2*^
Phase	1, 76	0.028	0.866	0.000
Condition	1, 76	0.078	0.781	0.001
Gender	1, 76	0.307	0.581	0.004
DMS	1, 76	5.975	0.017	0.073
Condition*Gender	1, 76	0.573	0.451	0.007
Condition*DMS	1, 76	0.120	0.730	0.002
Condition* Gender *DMS	1, 76	0.500	0.608	0.013
Phase*DMS	1, 76	1.426	0.236	0.018
Phase*condition	**1, 76**	**4.483**	0.038*	**0.056**
Phase * Gender	1, 76	0.425	0.516	0.006
Phase*Condition*Gender	1, 76	0.496	0.483	0.006
Phase*Condition*DMS	1, 76	1.708	0.195	0.022
Phase*Condition*Gender*DMS	1, 76	0.011	0.989	0.000

### Study 2 interim discussion

Overall, Study 1 findings showed some support for the visual diet hypothesis but the role of associative learning was less clear. Study 2 therefore involved introduced two further manipulation conditions, in which visual diet effects would be impossible, yet associative learning effects might still arise (equal exposure to muscular and non-muscular male bodies of differing valence). Study 2 data do show a significant interaction between test phase and condition such that Condition 6 manipulation stimuli significantly decreased preferences for muscularity but Condition 5 manipulation stimuli did not. Such findings suggest that associative learning mechanisms can, to some extent, underpin changes in body preferences for male muscularity. The Condition 6 findings specifically cannot be explained by the visual diet hypothesis, but rather must be the result of participants responding to the positive valence of the non-muscular male bodies and shifting their preferences accordingly (associative learning).

However, we note that viewing aspirational, high muscle mass males together with neutral low muscle mass males (Condition 5) did not increase preferences for muscularity as would be expected under the associative learning hypothesis. Rather, preferences stayed roughly the same following manipulation. Interestingly these findings are very similar to those reported in a previous paper where researchers report that aspirational large images together with non-aspirational thin images decreased preferences for thinness, whilst exposure to aspirational thin images together with non-aspirational large images resulted in no significant changes in preferences for thinness [17]. Our Condition 5 results may be due to the same phenomenon hypothesised in this previous paper [17]: perhaps there were no significant changes under Condition 5 because preference for muscularity was already high to begin with (pre-manipulation), because the Western sample used in the current study already inhabited an environment dominated by positive associations with muscularity in males. The aspirational high muscle mass trials therefore represented our sample’s pre-existing environment with limited scope for preferences for muscularity to increase any further post-manipulation.

Having said this, we found a significant interaction between phase, condition and gender and, when this interaction was explored further (by breaking data down into male and female data sets and analysing separately), results showed it was the male sample who provided more obvious evidence for associative learning mechanisms underpinning changes in body preferences. One explanation for this gender difference is social comparison processes [29], in which males identify with, preferentially focus upon, and compare themselves to, other males of a positive valence. If males preferentially focused on the positively valenced over the neutral images in each manipulation condition, this would explain why their preferences shifted in the direction of these aspirational/ positive valence manipulation images (as opposed to shifting in the direction of the neutral images) as predicted under the associative learning hypothesis. Additionally, aspirational images were retrieved from male clothing websites and thus the images were intended to be appealing to male (as opposed to female) viewers specifically which could, again, explain why associative learning effects were more pronounced for males in this case. Because stimuli was made up of male (vs female) bodies, the female sample were less likely than males to identify with, and thus attend to, aspirational manipulation stimuli. This may explain why associative learning effects were less pronounced for female participants. Consistent with Study 1 data and previous published findings [17] in which body concerns did not influence preference changes, findings from Study 2 showed that Drive for Muscularity scores did not influence the extent of changes in muscularity preferences following manipulation: analyses revealed no significant interactions between test phase, condition and Drive for Muscularity. We acknowledge, however, that participants completed the DMS at the end of the survey and exposure to stimuli in the many body preference trials may have temporarily affected participants’ body concerns. Indeed, exposure to muscular images can increase feelings of dissatisfaction following exposure [30, 31]. Asking participants to complete the DMS before the preference trials began may, in hindsight, have been a better method.

Whilst manipulation images were pre-rated for muscularity and valence in an attempt to select the most appropriate stimuli for Study 2, the raters were mostly (>80%) female (opportunity sample). It is worth noting, therefore, that these ratings may be biased towards the female perspective. We should not automatically assume that males would offer similar ratings here and therefore we recommend that those who replicate our work using the same stimuli should re-run the ratings task with more male raters to see if similar ratings of both muscularity and valence are obtained with such a sample.

## Study 3

As mentioned in our introduction, studies entailing repeated exposure to similar stimuli may result in demand characteristics, specifically participants discerning the intention of the study and consciously shaping their responses. With this in mind, a third study was conducted (Study 3) in which ‘distractor images’ were presented alongside the idealised manipulation images of bodies to lessen the potential influence of demand characteristics. Study 3 aimed to explore whether adaptation still occurred when manipulation image bias towards a particular body type was more subtle.

### Method

#### Participants

From an initial sample of 96 responses, 12 low quality participant responses were excluded from analysis for selecting the same response on all trials. This left us with a sample of 84 (45 men and 39 women) participants aged between 18 and 30 with a mean age of 25 (SD = 3.84) who were recruited for Study 3 through a participant recruitment website. 77% of the sample reported that they were exclusively heterosexual. Participants were randomly allocated to one of two manipulation conditions. Participants were told that the aim of the experiment was to explore ‘body preferences’. Participants received course credits where appropriate to thank them for their participation. The sample size for Study 3 exceeds the number of participants used in previous muscularity adaptation experiments (e.g. [21]). Further, sample size here exceeds the number of participants required for a two-way interaction using the power analysis run for Study 1.

#### Stimuli

Manipulation stimuli were made up of both photographs and CGI images. The photographs were the same as those used as neutral images in both Studies 1 and 2, whilst the CGI images used in manipulation as well as in both the pre- and post- preference for muscularity trials of Study 3 were created using DAZ Studio 4.10, using the ‘Genesis 2 Base Male’ in basic white briefs. These CGI images were created such that the high muscle mass CGI images were less muscular than those equivalent high muscle mass CGI images used in Study 1 and 2. This meant that any muscularity differences between high and low muscle mass CGI images in Study 3 were more subtle and reduced the likelihood of demand characteristics affecting results.

#### Procedures

Procedures largely mirrored those implemented in Studies 1 and 2: participants completed the pre-manipulation preference for muscularity task, followed by the manipulation phase and then the post-manipulation preference for muscularity task.

Participants were randomly assigned to one of two manipulation conditions. Condition A involved 41 (22 male and 19 female) participants viewing 48 (38 photographs and 10 CGI) high muscle mass images together with 22 (16 photographs and 6 CGI) low muscle mass male images. Condition B involved 43 (23 male and 20 female) participants viewing 48 (38 photographs and 10 CGI) low muscle mass images together with 22 (16 photographs and 6 CGI) high muscle mass male images. Images were presented in a randomised order.

### Results

**Fig 5:**
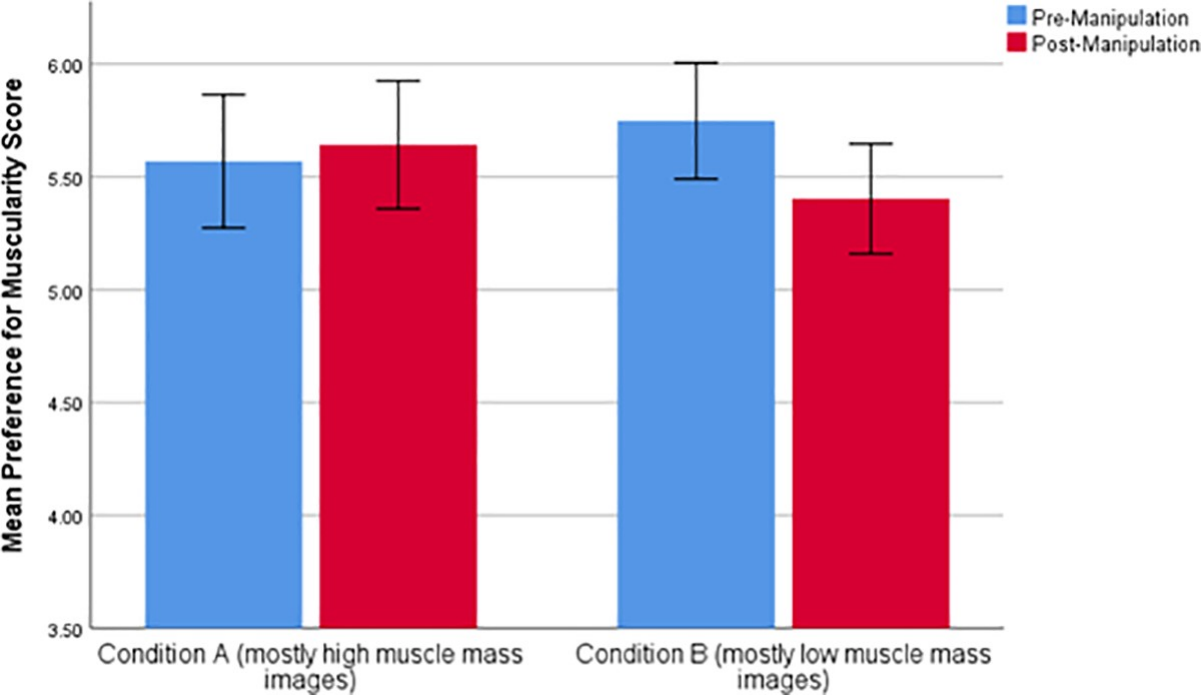
Mean changes in preference for muscularity following manipulation across each of the two conditions where 3.50 represents no preference to either image presented.

A mixed ANOVA with test phase (pre- versus post-manipulation) as a repeated measures variable and condition (Condition A versus Condition B) as the between-participants factor showed a significant interaction between test phase and condition (*F*1,82 = 9.612, p < .004, partial eta2 = .105) such that Condition A manipulation stimuli (48 high muscle and 22 low muscle mass images) increased preferences for muscularity and Condition B stimuli (48 low muscle and 22 high muscle mass images) decreased preferences for muscularity. Mean pre- and post-manipulation levels of muscularity preference for each condition are shown in Fig 5 and the tabulated values for the mixed ANOVA are presented in Table 10 under ‘Mode1’. There were no higher order interactions when gender was added to the model (see ‘Model 2’ in Table 10). A post-hoc paired-samples t-test revealed a significant difference between mean pre- and post-manipulation muscularity preference scores for condition B (*t*(42) = 3.299, *p* = .002), but no such significant difference exists under condition A (*t*(40) = .872, *p* = .389). The significant result for condition B survived when p values were corrected for multiple comparisons (using adjusted p = 0.025 for 2 tests).

**Table 10:** Tabulated values for mixed ANOVA with phase (pre- versus post- manipulation preference for muscularity scores) as the repeated measures variable and condition (condition A versus condition B) as the between-participants factor (Model 1) with gender added as an additional between-participants factor (Model 2). Critical tests of our hypotheses are shown in bold.

Source	*Df*	*F*	*p*.	*ηp* ^*2*^
**Model 1**				
Phase	1, 82	4.061	0.047	0.047
Condition	1, 82	0.290	0.865	0.000
Phase * Condition	**1, 82**	**9.612**	0.003**	**0.105**
**Model 2**				
Phase	1, 80	3.909	0.051	0.047
Condition	1, 80	0.025	0.875	0.000
Gender	1, 80	0.943	0.334	0.012
Phase*Condition	**1, 80**	**9.480**	0.003**	**0.106**
Phase*Gender	1, 80	0.016	0.899	0.000
Condition*Gender	1, 80	0.018	0.893	0.000
Phase*Condition*Gender	1, 80	0.096	0.757	0.001

### Study 3 interim discussion

Overall, Study 3 findings showed that when manipulation image bias is more subtle (i.e. when distractor images were presented alongside the manipulation images), preferences still shifted in the direction of the most prevalent (manipulation) image. This finding is of particular interest given the fact that most previous adaptation work has been designed with an obvious bias towards a particular image type as part of manipulation, rendering it vulnerable to the effects of demand characteristics [26]. Study 3 findings suggested that demand characteristics were unlikely to have confounded results in previous studies of adaptation as preferences for muscularity, in this study, shifted even when bias was so subtle that it was unlikely to be detected. Crucially, this mirrors the results of studies making use of manipulation imagery with an obvious skew towards a certain image type, thus suggesting that demand characteristics likely did not confound the findings of such studies, though we note that further replications of Study 3 are necessary to be more certain of this.

We note that Study 3 manipulation involved participants viewing images that were biased towards one particular body type of either high (Condition A) or low (Condition B) muscle mass. For example, 69% of manipulation images were of high muscle mass and 31% were of low muscle mass under Condition A (and vice versa in Condition B). Future work could explore how subtle this bias in image type can be in order for changes in body type preferences to still be observed.

## General discussion

The primary purpose of this research was to explore whether changes in preferences for muscularity in male bodies across a male and female sample was best explained by visual diet, associative learning mechanisms, or a mixture of the two. Study 1 provided evidence for the visual diet hypothesis, though the role of associative learning was less clear. Study 2 explored changes in muscularity preferences in a context in which visual diet effects would be impossible, yet associative learning effects could still arise. It showed that associative learning mechanisms also influenced changes in preferences for muscularity, in that exposure to aspirational non-muscular male bodies, alongside neutral muscular male bodies lead to a decrease in preference for muscularity. Phase and muscularity interactions were evident in every study.

This paper also explored whether demand characteristics were likely to have confounded the findings of previous adaptation experiments as proposed by previous authors in the field [26]. Study 3 showed that even when manipulation images are only subtlety skewed towards a particular body type (i.e. when potential demand characteristics are reduced), muscularity preferences still shifted towards the most prevalent image type shown as part of manipulation. This suggested that demand characteristics were unlikely to have confounded the results of previous adaptation experiments with more obvious manipulation image bias. Though, as previously mentioned, to be sure of this conclusion, replications are necessary.

CGI images used in the pre- and post-manipulation muscularity preference trials were highly controlled, with only muscularity manipulated. Although these images were very realistic, it would have been more ecologically valid to use real photographs naturally varying in muscularity or one photograph counter-manipulated in the muscularity dimension. This is something we would encourage others to consider when using similar experimental procedures in the future.

For Studies 1 and 2, both sets of neutral manipulation stimuli were photographs of nude males in the standard anatomical position with faces and genitals obscured, whereas faces were not obscured in the aspirational manipulation stimuli. Lack of consistency in facial cue availability is unlikely to have affected our results because eye tracking evidence shows that participants’ first fixations almost always land on the face, followed very quickly by fixations on the upper chest and pelvic regions of nude and clothed same and opposite sex human figures [32]. This, together with the fact participants were repeatedly instructed to compare each *body* to the one previously seen for every trial during the manipulation phase suggests the lack of consistency in facial cue availability across conditions is unlikely to be a confound. Having said this, we do acknowledge that to rule it out as a confound altogether we would need to re-run our studies either using eye tracking equipment such that we could confirm participants’ focus was on bodies or indeed ensuring all stimuli had faces obscured for consistency across the manipulation trials.

Previous eye tracking studies show that nude stimuli receive more fixations than clothed [32]. The nude (neutral condition) images used in in the current study may therefore be more salient than the clothed aspirational ones which may explain why the visual adaptation effect was particularly strong for Condition 4 (neutral, low muscle mass condition). Although if this was the case, we would also expect visual adaptation effects to be larger for Condition 3 (neutral, nude, high muscle mass) than for Condition 1 (aspirational, clothed, high muscle mass), as well as finding Condition 5 (aspirational clothed high muscle mass and neutral nude low muscle mass) to significantly decrease muscularity preferences following manipulation and Condition 6 (aspirational, clothed low muscle mass and neutral nude high muscle mass) to significantly increase them, yet we did not find this. Such findings therefore suggest nudity is unlikely to have confounded results. Having said this, as with facial cue availability, we cannot completely rule out nudity as a confounding factor. As such, future work may seek to explore whether findings replicate when the valence of imagery is altered through means other than high status clothing, for example, through varying health cues or facial expression perhaps.

Neutral manipulation images were not necessarily of a negative valence, for example, the neutral high muscle mass images were likely to be somewhat aspirational, given that muscularity on its own is an aspirational trait. This makes it difficult to compare the manipulation effects of neutral muscular images to the manipulation effects of aspirational muscular images (given that even such neutral images are somewhat aspirational). However, we considered this potential flaw prior to conducting Study 2 and thus ran a pre-rating task in which all stimuli used in Study 1 were rated in terms of how aspirational and how muscular each image was. For the Study 2 manipulation stimuli, the neutral images had mean valence ratings significantly lower than the mean valence ratings for the aspirational conditions as shown in Table 4. This means we had separate, clearly defined stimulus categories for high muscle mass and low muscle mass bodies that differed in terms of valence.

However, we also note (as shown in Table 4), that within the high muscle mass stimuli, the aspirational high muscle mass images had higher ratings of muscularity (mean value = 7.337) than the neutral high muscle mass images (mean value = 4.840). Mean values for muscularity within the low muscle mass stimuli were roughly the same, with a mean value muscularity score of 2.906 for the aspirational low muscle mass group and a mean score of 2.272 for the neutral low muscle mass group. Because the high muscle mass stimuli categories had different muscularity ratings across the aspirational and neutral high muscle mass conditions, this should be considered as a potential confound. Differences in such ratings may explain Study 1 findings in which participants’ preference for muscularity increased following exposure to aspirational muscular males but failed to show such an increase for neutral high muscle mass images (see Fig 2), in that adaptation to muscularity was stronger in cases where high muscle mass was more obvious. Further, the lower ratings of muscularity for the neutral high muscle mass stimuli may explain Condition 6 findings, in that these images were not sufficiently muscular to counter the effects of the aspirational non-muscular images. Having said this, high mean ratings of muscularity for the aspirational high muscle mass stimuli did not appear to counter the effects of neutral low muscle mass images in Condition 5. It is therefore unlikely that the differences in muscularity ratings across the aspirational and neutral high muscle mass groups are the primary reason for our findings. Further, whilst neutral high muscle mass images may have had lower muscularity ratings than the aspirational high muscle mass images, we argue that all high muscle mass images (whether neutral or aspirational/high valence) are of a more muscular physique than you would expect to see in most of our average raters/participants or their immediate social circle. Though we do note that future work in this area should make use of better matched stimuli for muscularity.

We recognise muscle mass and fat mass as having very different associations with health in males, yet being highly correlated (due to larger people having more fat and muscle). However, adaptation research has shown that fat and muscle mass are encoded separately because participants’ points of subjective normality shifted in the direction of the manipulation images along the relevant (fat or muscle) axis [21]. Although, the authors of this paper provide support for the visual diet hypothesis, they did not manipulate the valence of their stimuli and thus any associative learning effects were not clear. The current study has already explored the mechanisms underpinning changes in muscularity preferences using low muscle mass and high muscle mass male bodies (each with low fat mass) for stimuli, but we do not know whether associative learning effects are apparent using male body stimuli of differing BMI and valence and we therefore propose that this should be a future area of focus. Similarly, when exploring adaptation effects using female body stimuli, work has already explored the internal underlying mechanisms underpinning changes in female BMI preferences [17], however, future work should seek to explore whether such mechanisms also underpin shifts in preferences for muscularity using female body stimuli despite the fact that muscularity is an aspirational trait primarily associated with male bodies. Such findings will allow us to establish whether associative learning effects are only apparent when sex-specific body traits, such as high muscularity and low BMI, are assigned to the appropriate sex manipulation image bodies (e.g. high muscle mass males and low BMI females).

## Conclusion

In summary, our findings provide evidence for the visual diet hypothesis with some evidence for associative learning mechanisms. A primary implication of our findings is that media promotion of unrealistically muscular, unhealthily proportioned male bodies is likely to be increasing personal preferences for male muscularity in both men and women. High status male figures in the media are unrepresentatively muscular [19, 20], and exposure to such figures may be affecting perceptions of the ‘normal’ male body. Future work should build upon our current findings and should establish the foundations of mechanistic interventions to reduce the negative impact of ubiquitous hypermuscular male body images in the media.
